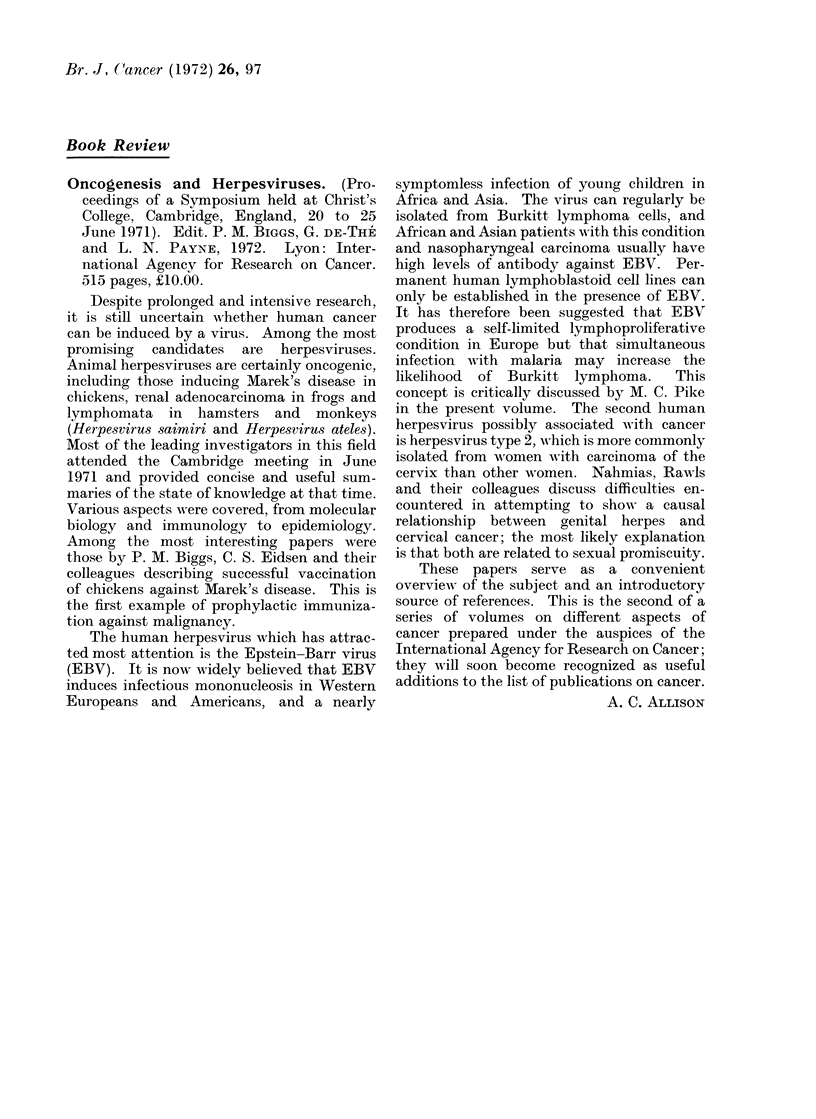# Oncogenesis and Herpesviruses

**Published:** 1973-01

**Authors:** A. C. Allison


*Br. J. Cancer* (1972) **26**, 97

### *Book Review*

**Oncogenesis and Herpesviruses.** (Proceedings of a Symposium held at Christ's College, Cambridge, England, 20 to 25 June 1971). Edit. P. M. BIGGS, G. DE-THÉ and L. N. PAYNE, 1972. Lyon: International Agency for Research on Cancer. 515 pages, £10.00.

Despite prolonged and intensive research, it is still uncertain whether human cancer can be induced by a virus. Among the most promising candidates are herpesviruses. Animal herpesviruses are certainly oncogenic, including those inducing Marek's disease in chickens, renal adenocarcinoma in frogs and lymphomata in hamsters and monkeys (*Herpesvirus saimiri* and *Herpesvirus ateles*). Most of the leading investigators in this field attended the Cambridge meeting in June 1971 and provided concise and useful summaries of the state of knowledge at that time. Various aspects were covered, from molecular biology and immunology to epidemiology. Among the most interesting papers were those by P. M. Biggs, C. S. Eidsen and their colleagues describing successful vaccination of chickens against Marek's disease. This is the first example of prophylactic immunization against malignancy.

The human herpesvirus which has attracted most attention is the Epstein–Barr virus (EBV). It is now widely believed that EBV induces infectious mononucleosis in Western Europeans and Americans, and a nearly symptomless infection of young children in Africa and Asia. The virus can regularly be isolated from Burkitt lymphoma cells, and African and Asian patients with this condition and nasopharyngeal carcinoma usually have high levels of antibody against EBV. Permanent human lymphoblastoid cell lines can only be established in the presence of EBV. It has therefore been suggested that EBV produces a self-limited lymphoproliferative condition in Europe but that simultaneous infection with malaria may increase the likelihood of Burkitt lymphoma. This concept is critically discussed by M. C. Pike in the present volume. The second human herpesvirus possibly associated with cancer is herpesvirus type 2, which is more commonly isolated from women with carcinoma of the cervix than other women. Nahmias, Rawls and their colleagues discuss difficulties encountered in attempting to show a causal relationship between genital herpes and cervical cancer; the most likely explanation is that both are related to sexual promiscuity.

These papers serve as a convenient overview of the subject and an introductory source of references. This is the second of a series of volumes on different aspects of cancer prepared under the auspices of the International Agency for Research on Cancer; they will soon become recognized as useful additions to the list of publications on cancer.

A. C. ALLISON